# Surviving the heat: heterogeneity of response in *Saccharomyces cerevisiae* provides insight into thermal damage to the membrane

**DOI:** 10.1111/1462-2920.12866

**Published:** 2015-05-14

**Authors:** Stéphane Guyot, Patrick Gervais, Michael Young, Pascale Winckler, Jennifer Dumont, Hazel Marie Davey

**Affiliations:** 1 UMR A 02.102 Procédés Alimentaires et Microbiologiques (PAM), Equipe Procédés Microbiologiques et Biotechnologiques (PMB)1 Esplanade Erasme, 21000, Dijon, France; 2 Spectral Imagerie Resource Center, Agrosup Dijon/Université de Bourgogne1 Esplanade Erasme, 21000, Dijon, France; 3 Institute of Biological, Environmental and Rural Sciences, Aberystwyth UniversityPenglais, Aberystwyth, Wales, SY23 3DA, UK

## Abstract

Environmental heat stress impacts on the physiology and viability of microbial cells with concomitant implications for microbial activity and diversity. Previously, it has been demonstrated that gradual heating of *Saccharomyces cerevisiae* induces a degree of thermal resistance, whereas a heat shock results in a high level of cell death. Here, we show that the impact of exogenous nutrients on acquisition of thermal resistance differs between strains.

Using single-cell methods, we demonstrate the extent of heterogeneity of the heat-stress response within populations of yeast cells and the presence of subpopulations that are reversibly damaged by heat stress. Such cells represent potential for recovery of entire populations once stresses are removed. The results show that plasma membrane permeability and potential are key factors involved in cell survival, but thermal resistance is not related to homeoviscous adaptation of the plasma membrane. These results have implications for growth and regrowth of populations experiencing environmental heat stress and our understanding of impacts at the level of the single cell. Given the important role of microbes in biofuel production and bioremediation, a thorough understanding of the impact of stress responses of populations and individuals is highly desirable.

## Introduction

*Saccharomyces cerevisiae* is used widely in both research and biotechnology and although it was the first eukaryotic organism to have its genome sequenced (Goffeau *et al*., 1996), 18 years later only 77.5% of the identified open-reading frames have an assigned biological function (http://www.yeastgenome.org/cache/genomeSnapshot.html 23 March 2015). Nevertheless, a range of genetic contributions to the fitness and survival of *S. cerevisiae* under sub-optimal conditions have been identified at the population level (Delneri *et al*., 2008; Davey *et al*., 2012; North *et al*., 2012). Notwithstanding these advances, heterogeneity in response to environmental challenges between individuals of a nominally clonal population is an important factor that is often overlooked, remains difficult to predict and yet has wide impacts on the economy of biotechnological processes, the interpretation of experimental results and the modelling of responses to environmental change (Davey and Kell, 1996; Avery, 2006).

Response to environmental changes is dependent first on sensing a shift away from previous conditions and, as the barrier between the interior and exterior of the cell, the membrane plays a role both in sensing change and in protecting the cell from it. Heat-induced stress leads to modification of the physical properties of the membranes of living cells. Lipids become more fluid as temperature increases (Chapman, 1975) and, if the temperature increases beyond the tolerance limit of the organism, this can lead initially to increased membrane permeability, followed by disorganization of the lipid/protein membrane order and finally to a loss of membrane integrity and cell death (Hazel, 1995; Los and Murata, 2004).

Psychrophilic organisms are able to synthesize proteins at −20°C (Junge *et al*., 2006), whereas hyperthermophiles are able to divide (and hence synthesize proteins) at temperatures higher than 120°C (Kashefi and Lovley, 2003). Differences in membrane lipids and stabilizing substances are common between species adapted for these extreme environments. Eukaryotic microorganisms are more modest in their temperature range, being reported to be physiologically active from −5°C (Ward, 1966) to approximately 60°C (Maheshwari *et al*., 2000). *Saccharomyces cerevisiae* is reported to be capable of growth at temperatures ranging from 1°C to 46°C with a strain-dependent optimum in the region of 30°C (Salvado *et al*., 2011). However, in the right environment, at least a proportion of the cells will retain viability outside of this range – this is well characterized in culture collections stored at −80°C in cryopreservants. At higher temperatures, cultivability has been shown to be retained in cells exposed to temperatures of 55°C providing that the culture is returned to a more suitable temperature for observation of growth (López-Malo *et al*., 1999). Survival of small numbers of cells may be a consequence of shielding within groups and indeed stressful environments may be a driver for multicellularity in predominantly unicellular organisms (Ratcliff *et al*., 2012).

In natural environments, fluctuations in temperature on a diurnal and/or seasonal basis are commonplace (Tai *et al*., 2007). Aside from its role in the laboratory, brewery and winery, the environmental niche of *S. cerevisiae* is considered to be plant surfaces (Mortimer and Johnston, 1986; Mortimer and Polsinelli, 1999) and cells may be expected to adapt to temperature changes to accommodate fluctuations in their natural environment. Heterogeneity, characterized more recently as bet-hedging, is a potential survival strategy for organisms that cannot control their external environment, particularly where stresses are varied and intermittent (Levy *et al*., 2012; Hellweger *et al*., 2014). Differences in cell cycle position, cellular clocks, individual cell history and random stochastic events all contribute to different levels of resistance in a nominally clonal population. Within limits, when organisms are exposed to hyperthermic conditions, they are able to respond in a variety of ways (other than survival versus death) including active modification of the chemical composition of the lipid component of the membrane, expression of stress proteins and/or synthesis and accumulation of a variety of solutes (e.g. trehalose), which maintain their lipids in a functional state (Horvath *et al*., 1998; Singer and Lindquist, 1998; Coucheney *et al*., 2005).

The effects of hyperthermia on the plasma membrane have been investigated by comparing the membrane characteristics of thermally adapted cells with those of a control population (Swan and Watson, 1997). For practical reasons, comparatively few studies have focused on precisely how the plasma membrane changes during the exposure of cells to perturbation. However, there are two major drawbacks to assessing the impact on populations of adapted cells. First, cells respond on an individual rather than a population basis, and survival of a population of adapted cells may be a consequence of regrowth of a small number of survivors. Second, the kinetics of the temperature variation rate have also been shown to influence survival in many cell types (Kaur *et al*., 1998; Martinez de Marañon *et al*., 1999) with thermal shocks being more detrimental than gradual variation of temperature (heat slope) over the same range (Guyot *et al*., 2005; 2010[Bibr b16],[Bibr b17]). Rapid cooling applied immediately after the heat slope eliminates thermal resistance, suggesting a role for plasma membrane phospholipids via reversible and temperature-dependent phase transitions (Guyot *et al*., 2005).

The aim of this work was thus to characterize heterogeneous, asynchronous populations of yeast to determine the impacts of the heat-stress kinetics on the plasma membrane physiology both at the single-cell level and at the population level in two different genetic backgrounds. BY4741 is a well-characterized laboratory strain and CBS1171 is an industrial strain. CBS1171 has recently (Zhou *et al*., 2014) been compared with S288C (the parental strain of BR1171). The industrial strain has been shown to have differ particularly with respect to MAPK- and phosphatidylinositol-signalling pathways with upregulated expression of these genes in response to chemical stress. To improve understanding of the role of the plasma membrane, we have firstly investigated the evolution of its physico-chemical properties (permeability and potential) over a heat slope and a heat shock after recovery at 25°C with regard to unstressed samples (i.e. control). As the results showed a great difference in the behaviour of the membrane between gradually heat-stressed samples and those exposed to a heat shock, we also investigated fluidity changes in the membrane on a population level and at the single-cell level after recovery at 25°C. The results presented contribute to our understanding of the potential for microbes to adapt to temperature fluctuations and extremes in the natural environment.

## Results

### Acquisition of thermal resistance is a strain-dependent process

**Figure 1:**
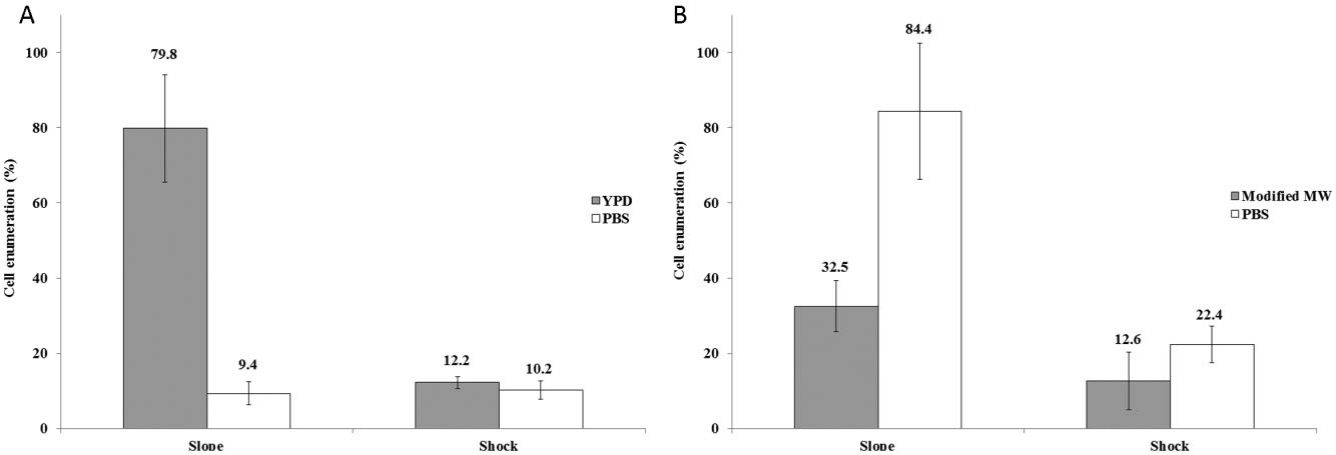
Yeast cells were exposed to a heat slope or heat shock as described in the Experimental procedures. The number of colonies obtained following treatment is expressed as a percentage of the unstressed control. Error bars represent 95% confidence intervals.A. BY4741.B. CBS1171.

We first determined whether the availability of nutrients in the suspending medium had an impact on the loss of cultivability of the strains BY4741 and CBS1171 exposed to a heat slope and a heat shock. Figure 1A shows that for the strain BY4741 where the suspending medium is devoid of nutrients (phosphate-buffered saline, PBS), there is no acclimation observed during the heat-slope treatment. However, when the cells are suspended in yeast peptone dextrose (YPD) growth medium prior to and during exposure to a heat slope, there is an acquisition of thermal resistance that is not observed with heat-shocked cells. In strain BY4741, the acquisition of resistance to the elevated temperature required the presence of nutrients and is therefore an active metabolic process. Interestingly, as shown in Fig. 1B and as will be discussed later, this finding differs from the behaviour of strain CBS1171, which can acquire thermal resistance in the absence of nutrients (i.e. in PBS) when exposed to a heat slope. Surprisingly, in the absence of nutrients, the degree of thermal resistance is higher than in their presence modified Malt Wickerham medium (modified MW). The results presented here lend weight to the hypothesis that acquisition of resistance is a complex strain-dependent process in which nutrients play a key role.

### Impact of thermal stress on membrane integrity

**Figure 2:**
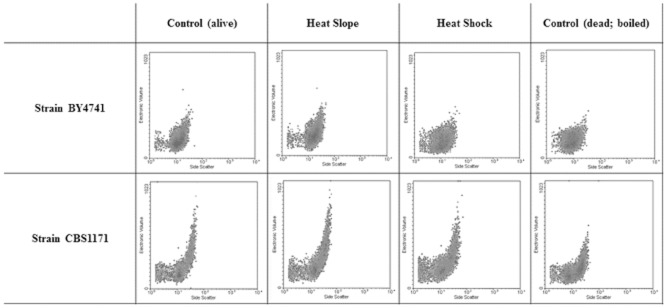
Flow cytometric analysis of electronic volume and side scatter of two yeast strains exposed to different heat stresses.

Previous publications (Gervais and Martínez de Marañón, 1995; Morozov *et al*., 1999; Marechal *et al*., 1999; Martinez de Marañon *et al*., 1999) concerning strain CBS1171 demonstrated the consequences of heat slope and shock on the reduction in colony-forming units. More recently, Guyot and colleagues (2005) showed that the heat-slope-induced thermal resistance could be eliminated by a rapid cooling from 50°C to 25°C, meaning that reversible and thermally dependent mechanisms were related to this type of resistance. Flow cytometry was used to characterize single cells, and data can be misleading if clumping of cells occurs, as this leads to events representing a number of cells rather than individuals, although it should be noted that the same issue compromises plate count analysis. To ensure that this was not a major problem in the current study, the electronic volume and side scatter signals were examined. Measurements were performed after recovery at 25°C, and results presented in Fig. 2 show that although there is greater heterogeneity in the electronic volume signal in CBS1171 than in BY4741, even the most extreme heat treatment (boiling) does not increase the heterogeneity or shift the population to higher channel numbers.

**Figure 3:**
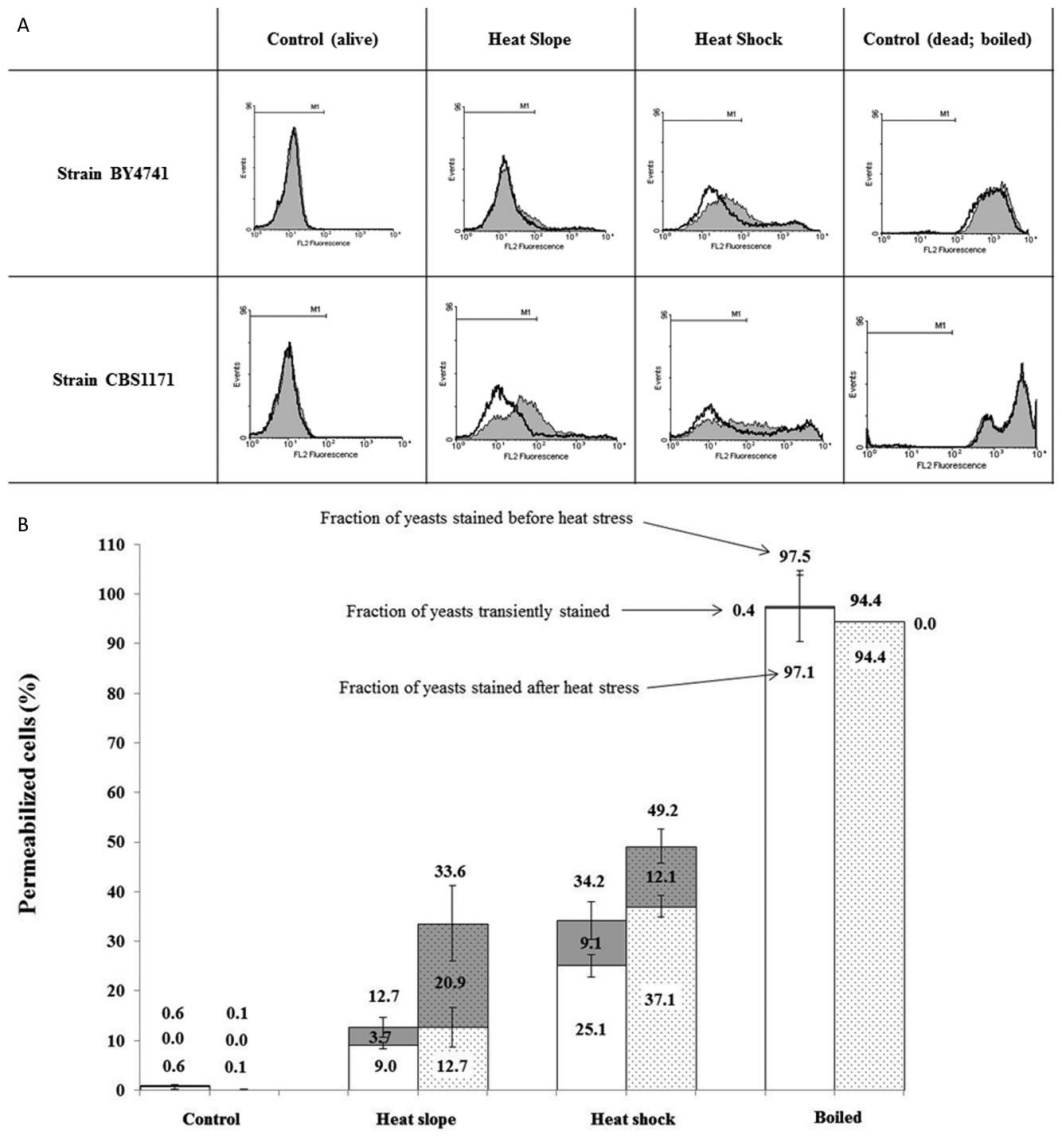
Flow cytometric analysis of yeast cells stained with propidium iodide as described in Experimental procedures.A. M1 is the region where the fluorescence signal is negative (determined using live cells plus PI). Shaded histograms represent samples in which the PI was added before the heat stress was applied, whereas the black lines represent samples cooled to room temperature prior to addition of the stain.B. To evaluate the fraction of transiently permeabilized yeast cells, the probe was added before or after the heat stress. The fraction of transiently positively stained yeasts corresponds to the difference between the fraction positively stained when PI was added before and after the stress. The first set of histograms (unshaded) represents BY4741 strain and the second set (shaded) represents CBS1171 strain. The fraction of yeasts stained positively corresponds to those outside of the M1 region (see Fig. 3A). Error bars represent 95% confidence intervals.

Flow cytometric monitoring of propidium iodide (PI) uptake is a widely used, rapid method for monitoring cell death based on the assumption that the intact membrane of a viable cell excludes the propidium ion and that loss of this permeability barrier represents irreparable damage and thus cell death. However, transient permeability of the cell membrane in stressed cells has been reported (Shi *et al*., 2007; Davey and Hexley, 2011), and consequently, we took the approach previously outlined (Davey and Hexley, 2011) of comparing samples exposed to thermal stress in the presence of PI with samples exposed to stress and then returned to room temperature before addition of PI. Figure 3A shows clear separation of the live control from the dead (boiled) control for both strains of yeast, confirming that extreme thermal stress damages the membrane barrier. It is also evident that the heat-slope regime causes damage to fewer cells than is observed with heat shock. Furthermore, there is a difference between the two strains studied with CBS1171 showing greater sensitivity to the heat stress than BY4741. When the consequence of addition of PI during or after the heat stress is considered, a shift in the population to higher fluorescence intensity is seen (Fig. 3A) for both strains when PI is present during the heat shock. BY4741 was more resistant to the heat slope than CBS1171 and there was little difference between the fluorescence profile of the BY4741 populations exposed to PI during or after the heat slope. However, although the majority of CBS1171 cells remain in the M1 region (Fig. 3A), they are shifted to a higher fluorescence. Figure 3B indicates the impact of the different heat treatments on the two strains of yeast, showing the greater sensitivity of CBS1171 (shaded bars) to both heat slope and heat shock when compared with BY4741 (unshaded bars). There is also evidence of higher transient permeability in CBS1171 during the heat-slope treatment.

### Impact of thermal stress on membrane polarization

**Figure 4:**
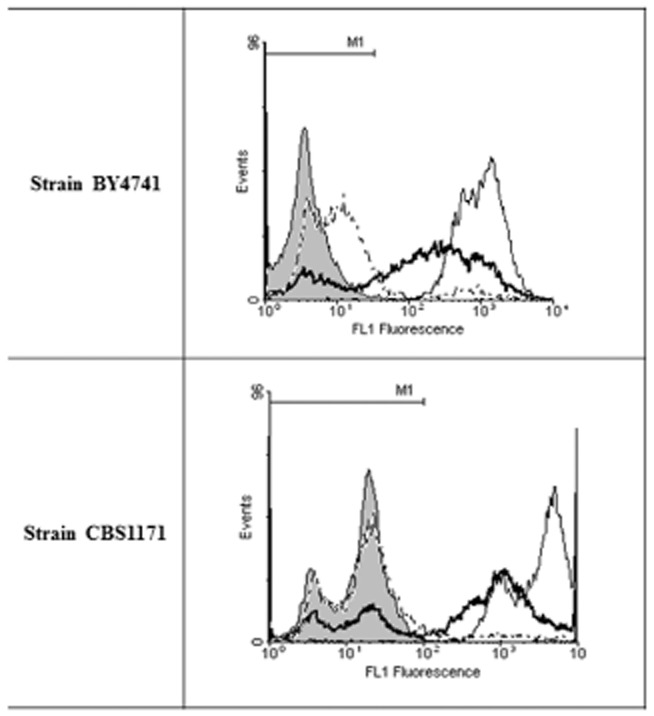
Flow cytometric analysis of yeast cells stained with DiBAC_4_(3) as described Experimental procedures. M1 is the region where the fluorescence signal is negative (determined using live cells plus stain). Shaded, control; dotted, heat slope; thick line, heat shock; thin line, boiled.

In comparison with propidium iodide where significant membrane damage is needed to allow entry into cells, the uptake of the anionic oxonol dye [DiBAC_4_(3); bis(1,3-dibutylbarbituric acid)trimethine oxonol] occurs with less-damaged cells and increases with depolarization of the cell membrane (Davey *et al*., 2004). Figure 4 shows the extent of dye uptake in yeast cells exposed to the different intensities of heat stress. There is clear separation between live (unstressed) and dead (boiled) cells. Although there are some changes in the shape of the population profiles, fluorescence intensity of yeast exposed to a heat slope remains largely within the range of channel numbers occupied by the unstressed control. In contrast, samples exposed to a heat shock have a higher proportion of cells with depolarized membranes (Table 1).

**Table 1:** Flow cytometric analysis of plasma membrane potential using DIBAC4(3)

Strain	Control	Heat slope	Heat shock	Boiled
BY4741	2.39 ± 0.66	13.17 ± 2.78	70.62 ± 2.11	98.64
CBS1171	1.98 ± 1.13	11.73 ± 0.56	62.50 ± 3.44	95.92

Values indicate percentage of the population that is positively stained.

### Absence of homeoviscous adaptation of the plasma membrane during the heat slope

**Figure 5:**
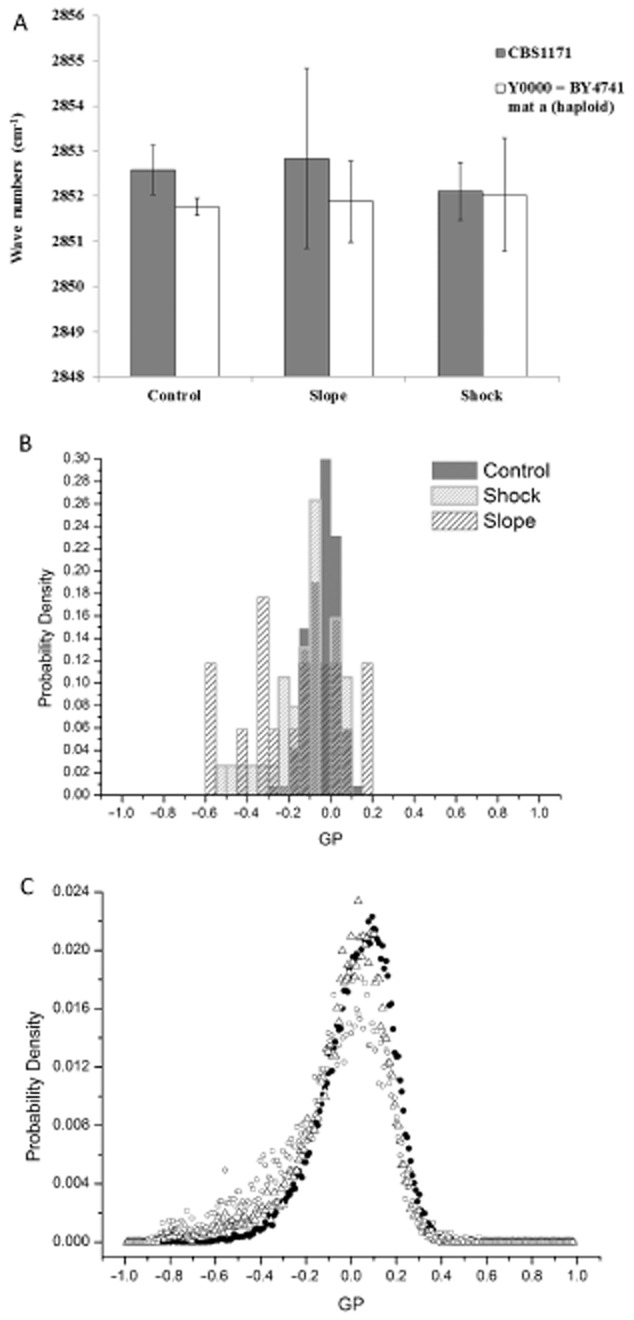
Evaluation of plasma membrane fluidity after recovery at 25°C. Measurements were performed after heat stress as described in Experimental procedures using (A) FTIR spectroscopy and (B–C) the hydrophobic fluorescent probe Laurdan.A. *FTIR* *measurements*. The (*ν*)CH_2_ symmetric stretching band (located around 2852 cm^−1^) of yeast cells of *Saccharomyces cerevisiae* (■) CBS1171 and (□) BY4741 was measured after recovery at 25°C (see Experimental procedures for details).B–C. *Two-photon imaging*. Yeast cells of *Saccharomyces cerevisiae* BY4741 were labelled with the hydrophobic fluorescent probe Laurdan after recovery at 25°C. Frequency distribution of GP values measured (B) at the level of the single cell and (C) at the level of the entire population were plotted (see Experimental procedures for details) for a representative experiment (control and heat-stressed samples were prepared from the same culture). Legend for (B) is given in the key. Legend for (C) is (●) control, (Δ) shock, (○) slope.

The maintenance of the correct degree of cell membrane fluidity is critical for cell function and integrity. Generally, there is an increase in saturated fatty acid content at higher temperatures and this compositional adaptation of membrane lipids, called homeoviscous adaptation, serves to maintain the correct membrane fluidity in the new conditions (Los and Murata, 2004). Accordingly, at 50°C the plasma membrane of thermal-resistant yeasts (i.e. previously exposed to the heat slope) might be predicted to be less fluid than that of heat-shocked yeasts to maintain membrane functionality. To determine whether the basis of acquisition of thermal resistance was a consequence of changes in membrane structure or composition, changes in plasma membrane fluidity of unstressed and heat-stressed cells (analysed after recovery at 25°C for technical reasons, see Experimental procedures section) were monitored using (i) infrared spectroscopy (Fourier transform infrared or FTIR: Fig. 5A) and (ii) the hydrophobic fluorescent probe Laurdan (two-photon fluorescence imaging; Fig. 5B). FTIR allowed us to analyse membrane fluidity at the population level, whereas Laurdan probe allowed us to obtain information both at the single-cell and at the population levels.

#### FTIR measurements

FTIR spectroscopy was used to monitor the disorder of the acyl chains of lipids via the frequency of the symmetric CH_2_ stretching mode (∼ 2852 cm^−1^) (Los and Murata, 2004). This frequency decreases as the membrane lipids transition from an ordered to a disordered state. The measurements presented in Fig. 5A were performed on strains CBS1171 and BY4741. After brief recovery at 25°C (see Experimental procedures), there was no significant difference (i.e. *P*-value >0.05) for either strain (CBS1171 and BY4741) between control and heat-stressed samples (slope and shock).

#### Two-photon fluorescent probe imaging

Heat-stressed (slope and shock) yeast cells of BY4741 strain were labelled with the hydrophobic fluorescent probe Laurdan after recovery at 25°C. For comparison, unstressed yeasts (control maintained at 25°C) were labelled with the same probe. This approach permitted analysis of plasma membrane fluidity both at the level of the entire population and at the level of the single cell (see Experimental procedures for details). Frequency distribution of the generalized polarization (GP) recorded both at the single-cell level (Fig. 5B) and at the entire population level (Fig. 5C) shows no difference between samples exposed to heat stress (following return to 25°C) and their corresponding control.

From these experimental results and because of the reversibility of mechanisms involved in this type of resistance, it can be concluded, both at the level of the entire heterogeneous population (which by the end of the heat-stress experiment contained a mixture of live, damaged and dead cells) and at the level of the single cell, that heat-slope-induced thermal resistance is not a consequence of differences in the plasma membrane lipid composition occurring during the heat slope.

## Discussion

Although eukaryotic cells can respond to mitigate the effects of an increase in environmental temperature (Chatterjee *et al*., 2000; Jenkins, 2003), sub-lethal high temperatures have nevertheless been shown to have a number of detrimental effects. Here we concentrate on effects of heat stress on the so-called ‘boundary of life’; the plasma membrane, as the essential barrier between the living cell and the exterior medium. In extreme conditions, heat may render a whole population non-viable. More commonly in the natural environment at least, heat stress will lead to loss of cultivability in a proportion of the cells, whereas others survive (although they may be damaged and require a period of recovery before resuming growth). Consequently, methods such as flow cytometry that quantify the heterogeneity of the sample are of particular value in these investigations (Kell *et al*., 1991; Dinsdale *et al*., 1995; Lloyd and Hayes, 1995; Davey and Kell, 1996; Lloyd and Dinsdale, 2000; Shapiro, 2003).

In the work presented here, we demonstrate (Fig. 1) that a proportion of the BY4741 and CBS1171 yeast cells in a population can survive a heat stress of 50°C and proceed to form colonies on an agar surface. This proportion increases if the heat stress is applied gradually, giving time for acclimatization, but the role of nutrients is different in the two strains. Nutrients appear to be required for the acquisition of thermal resistance in the BY4741 strain, whereas they reduce this type of resistance in CBS1171. In the absence of nutrients, the nature of solutes also influences cultivability as the CBS1171 strain exhibits a higher level of cultivability after a heat shock at 50°C for 30 min in a binary water-sorbitol medium (Martinez de Marañon *et al*., 1999: ∼ 60%) than in PBS (present study: ∼ 22%). Nevertheless, based on these studies after a heat slope followed by a 30 min plateau phase at 50°C, similar cultivabilities are observed in both conditions (around 80%). We therefore surmise that acquisition of protection from the heat stress is an active process that may be fuelled by endogenous nutrients in some cases. However, we do not exclude inter-strain differences as an alternative explanation. This knowledge has implications for methods used for eradication of viable cells as well as for maintaining the viability of cells during biotechnological exploitation and provides insights into survival of individuals within populations during environmental stress.

In order to improve characterization of the role of the plasma membrane in this type of resistance, we have investigated the measurement of its physico-chemical properties after recovery at 25°C (for technical reasons) using flow cytometry (permeability and potential) and FTIR/Laurdan probe (fluidity). Laurdan was developed for estimation of polarity in proteins (Macgregor and Weber, 1986), but as a polarity-sensitive probe, it has been used more generally including for measurements of lipid bilayers (Bagatolli, 2003). It has a blue emission in ordered lipid phase that is red-shifted as the lipid phase becomes disordered. Thus, a shift in the GP value (see Experimental procedures) would indicate a change in membrane structure.

Flow cytometric analysis performed after recovery at 25°C reveals differences between the two strains of *S. cerevisiae* studied. The membrane of BY4741 appears to retain its integrity (PI exclusion) better than that of CBS1171 in response to a heat slope, but there is a clearer increase in mean fluorescence intensity of BY4741 cells stained with DiBAC_4_(3) although the signal for the majority of cells remains within the range encompassed by unstressed cells. This increase in DiBAC_4_(3) signal, that is of a lower magnitude than is observed with dead cells, points to cells beginning the ‘journey from life to death’ (Davey, 2011) and flow cytometry with fluorescence-activated cell sorting may permit future characterization of the distance that can be travelled before the destination is decided. On entry into depolarized cells, DiBAC_4_(3) binds to intracellular proteins or membranes and the resulting fluorescence is enhanced and red-shifted (Epps *et al*., 1994). DiBAC_4_(3) is excluded from mitochondria and so changes in the signal provide information on the plasma membrane potential. Further work may reveal whether this is representative of adaptation of the cells within limits to the gradual temperature increase and that via an associated process, the membrane integrity is maintained. In the case of the heat slope, there was little difference for PI-staining of BY4741 cells irrespective of whether PI was present during or only after heat stress. This demonstrates that there was little or no transient permeabilization of these cells in this case. However, with strain CBS1171, there is a clearly observable shift to higher fluorescence intensity when PI is present during the heat stress (Fig. 3A), showing that in this case, transient permeabilization does occur. There is also a larger difference between CBS1171 cells exposed to the heat-slope and the unstressed control, demonstrating that this strain is less resistant to heating even with the more gradual kinetics of a heat slope. From these observations, one can ask whether plasma membrane fluidity and so homeoviscous adaptation of the membrane could be responsible for heat-slope-induced thermal resistance.

We therefore investigated whether plasma membrane fluidity of heat-treated cells (slope/shock) was a consequence of changes in membrane composition during the thermal treatment (Fig. 5). Our observations strongly suggest that no homeoviscous adaptation of the plasma membrane occurred during the 30 min plateau phase following the heat slope or the heat shock. Previously, it has been shown that the thermal resistance acquired during the heat slope could be eliminated by a rapid cooling from 50°C to 25°C, implying that rapid reversible and thermally dependent mechanisms are involved (Guyot *et al*., 2005) rather than substantial, permanent adaptation. However, these methods cannot exclude changes in plasma membrane composition during the brief cooling step (i.e. centrifugation at 25°C before FTIR measurements or analysis at 25°C using the two-photon microscope).

Our observations show that after recovery at 25°C, functional properties of the plasma membrane (characterized through the membrane potential) were similar between unstressed yeasts (i.e. control maintained at 25°C) and yeasts previously exposed to the heat slope, whereas a great difference was recorded between yeasts previously heat shocked and these previously exposed to the heat slope. Complementary results show that plasma membrane fluidity did not change with regard to unstressed yeasts after recovery at 25°C whatever the heat kinetics previously applied. In light of these observations, one can suggest that the nature of the modifications of the plasma membrane occurring during the heat slope is not related to the presence of *de novo* phospholipids (integrated into the membrane from newly synthesized or the basal pool of phospholipids) or other molecules which would have the capacity to modify membrane fluidity. It seems more feasible that a modification of the phospholipid chemical structure occurs that can be changed rapidly by specific enzymes such as lipases and saturases during the heat slope as well as during the cooling step.

In summary, although we demonstrate that homeoviscous adaptation is unlikely as an explanation for the observations, we present data supporting other mechanisms related to the behaviour of the plasma membrane including transient versus irreversable permeability and loss of membrane energization. In order to try to rationalize the basis of the difference between the two strains, future work should consider the role of proteins, protectants such as trehalose (Levy *et al*., 2012) and growth rate (Hellweger *et al*., 2014). These findings also have a number of practical implications. First, they add further weight to the argument (Davey and Hexley, 2011) that care must be taken regarding the synonymous use of the terms ‘PI-positive’ and ‘dead’. Second, the differences in loss of cultivability and transient permeabilization of two well-characterized laboratory strains of *S. cerevisiae* when exposed to heat stress illustrate the potential pitfalls of applying published methods and assumptions to new scenarios. Third, the data provide evidence for higher levels of transient permeability than previously reported and this may have a role to play in horizontal gene transfer (Davey and Hexley, 2011). Finally, the methods presented here provide a novel platform for assessing the impact of changes in temperature on individual cells within a population, and it is these measurements that may be expected to underpin further elucidation of the mechanisms of survival and death in cells exposed to environmental stress.

## Experimental procedures

### Yeast strains and cultivation

Two strains of *Saccharomyces cerevisiae* were used in this study: (i) the strain CBS1171 and (ii) the strain BY4741 *Δho*. Strain CBS1171 was grown on a modified MW medium composed of 10 g of glucose (Sigma Aldrich, Saint Quentin Fallavier, France), 3 g of pancreatic peptone (VWR International, Limonest, France), 3 g of yeast extract (Sigma Aldrich) and 1.5 g of NaH_2_PO4 (VWR International) in 1 L of distilled water. Based on previous work with this strain for heat stress studies (Guyot *et al*., 2005), a pre-culture was prepared by introducing one colony grown on solid medium [modified MW with 20 g l^−1^ agar (VWR International)] into a 250 ml conical flask containing 100 ml of medium and shaken at 250 r.p.m. for 48 h at 25°C on a rotary shaker. Then 100 μl of the pre-culture was inoculated into a 250 ml conical flask containing 100 ml of medium and shaken at 250 r.p.m. for 48 h at 25°C. Cultures were grown to reach the stationary phase.

BY4741 *Δho* knockout strain is from a Mat*a* haploid yeast deletion collection with the following background: *MATa his3Δ1 leu2Δ0 met15Δ0 ura3Δ0 YDL227c::kanMX4*. The *ho* gene encodes an endonuclease responsible for initiating mating-type switching, and BY4741 *Δho* is considered as a pseudo-wild type reference strain (Oliver *et al*., 1998). This strain was grown on YPD medium composed of 10 g of yeast extract (LabM, Lancs. UK), 20 g of bacteriological peptone (Oxoid, Basingstoke, UK) and 20 g of D-glucose (Fisher Scientific, Loughborough, UK) dissolved in 1 L of distilled water. One colony grown on medium solidified with 2% Agar (LabM) was introduced into a 250 ml conical flask containing 100 ml of liquid medium and shaken at 200 r.p.m. for 48 h at 25°C. Cultures were grown to reach the stationary phase.

### Evaluation of plasma membrane fluidity

#### FTIR

The changes in membrane fluidity were estimated using FTIR spectroscopy. Spectra were measured using hydrated (and not dried) samples. Five 100 μl samples from the same culture were pooled immediately after the heat treatment and were centrifuged at 2880 × *g* for 5 min at 25°C (the corresponding control samples from the same culture were pooled and centrifuged using an identical protocol). The yeast pellets were then placed on an infrared-transparent ZnSe window and equilibrated for 2 min at 25°C. The temperature was regulated by water circulation in a double envelope surrounding the window. The attenuated total reflectance FTIR second-derivative spectra were recorded between 4000 and 900 cm^−1^ (wavenumbers) on a Vector 22 FTIR spectrometer from Bruker (Karlsruhe, Germany) with a Bio-ATR II unit and equipped with a mercury–cadmium–telluride detector. The spectral resolution was 4 cm^−1^. To obtain the spectrum for each sample, 10 scans were recorded and analysed using the opus 6.5 software (Bruker). A standardized approach to data preprocessing was used to analyse the FTIR spectra. Data preprocessing first included a smoothing of the spectrum (17 smoothing points, provided by the opus 6.5 software) and then a second-derivative transformation. The peaks of interest were determined using the opus 6.5 peak-picking function: only peaks with negative intensities were considered ‘true’ peaks. Variations in plasma membrane fluidity were evaluated by measuring the vibrational modes of the (*ν*)CH_2_ symmetric stretching band located around 2850 cm^−1^ (Leslie *et al*., 1994).

#### Two-photon fluorescence imaging

Cells at an average concentration of 2.10^6^ cells ml^−1^ were labelled after recovery at 25°C with Laurdan (Sigma Aldrich) at a final concentration of 10 μM in dimethylsulphoxide (DMSO, Sigma Aldrich), and immediately imaged to avoid excess internalization of the fluorescent probe. The corresponding control was labelled using the same protocol.

Laurdan GP imaging was done using two-photon excitation. Imaging was carried out on a Nikon A1-MP scanning microscope (Nikon, Japan) with a ×60 Apo infrared (IR) objective (NA: 1.27, Water Immersion, Nikon, Japan). Excitation at 800 nm was provided by an IR laser (Chameleon, Coherent). The red and blue components of Laurdan fluorescence were split into two channels by using a dichroic beam splitter (DI01-R442, Semrock). Two optical band-pass filters (FF01-494/20, FF01-445/20, Semrock) with 20 nm bandwidth and centres at 445 and 494 nm were used to collect fluorescence in the blue and red regions of the Laurdan emission spectrum. The two ssimultaneous 512 × 512 pixels images obtained from the sample were processed applying the GP formula described by Equation 1 (where *I*_445_ and *I*_494_ refer to the average emission intensities at the specified wavelengths [subscripts]) to each pixel, using the generalized polarization analysis Image J plugin (Owen *et al*., 2012). Plasma membrane fluidity increases with GP decrease.

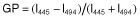
1

Two methods were utilized to analyse GP value: (i) analysis at the population level: study of the frequency distribution of GP of all pixels co-located with the plasma membrane of yeasts (several photos per sample), (ii) analysis at the single-cell level: the mean GP value of the surrounding plasma membrane per cell was calculated and then the frequency distribution of means collected for every yeast per sample was plotted. The two methods were applied to the same photos and so to the same samples. Because Laurdan is a hydrophobic probe, it is rapidly internalized into intracellular membranes, but the imaging approach ensured that only fluorescence of the plasma membrane was taken into account (530 nm width).

### Preparation of samples for flow cytometry and enumeration

#### Preparation of samples

One millilitre of yeast culture was placed into a 1.5 ml microcentrifuge tube and centrifuged for 5 min at 2880 × *g* at room temperature. The cell pellet was resuspended in 1 ml of fresh growth medium (modified MW for CBS1171 strain or YPD for BY4741*Δho* strain) or non-nutritive medium (PBS), vortex-mixed and finally 100 μl of this suspension was introduced into a 200 μl PCR microtube.

#### Thermal treatments

Samples (100 μl) were heat treated using a PCR gradient thermal cycler (MJ Research PTC200, MJ Research, Waltham, USA). Yeast suspensions were first maintained for 5 min at 25°C and then exposed either to (i) a heat slope or (ii) a heat shock from 25°C to 50°C, both followed by a 30 min plateau phase at 50°C. The heat slope was applied at a rate of 0.5°C min^−1^, whereas the heat shock was applied within 36 s (heating rate was approximately 33.3°C min^−1^).

### Cell enumeration

Yeast concentration was estimated using the colony forming unit method by plating 10 μl replicates of the appropriate decimal dilution of the cell suspension on solid medium (YPD-agar for BY4741 *Δho* strain and modified MW-agar for CBS1171 strain) immediately after the heat stress had been applied (or immediately after washing samples with fresh liquid medium in the case of control samples).

### Preparation of fluorescent stains

Propidium iodide (Sigma Aldrich, Dorset, UK) was used at a final concentration of 6 μg ml^−1^ from a stock solution of 606 μg ml^−1^ or 12 μg ml^−1^ in PBS (Sigma Aldrich) prepared in Millipore Milli-Q (0.22 μm filtered) water. The stock solutions were stored at −20°C.

Anionic bis-(1,3-dibutylbarbituric acid) trimethine oxonol also named bis-oxonol (DiBAC_4_(3)) (Sigma Aldrich) was used at a final concentration of 5.7 μM from a stock solution of 1 mM in water. The stock solution was stored at −20°C.

### Flow cytometry

Flow cytometric analyses were performed using a Cell Lab Quanta Flow cytometer (Beckman Coulter, UK). Measurements were made using the integrated Coulter electronic volume detector as well as side scatter from the argon ion (488 nm) laser. Fluorescent probes were also excited with the 488 nm laser. The propidium iodide and the DiBAC_4_(3) fluorescence were respectively collected via a 575 nm band-pass filter and a 525 nm band-pass filter. A total of 10 000 events were collected and analysed using Windows Multiple Document Interface for Flow Cytometry (WinMDI 2.9).

Plasma membrane permeability was identified using the non-permeant propidium iodide probe. In order to evaluate the proportion of cells transiently permeabilized during the heat treatment, some samples were stained during or after the heat treatment as previously described (Davey and Hexley, 2011).

To stain samples before the thermal treatment, propidium iodide was added to the 100 μl of yeast suspension that had been introduced into a 200 μl PCR microtube. To avoid unnecessary dilution 1 μl of propidium iodide stock solution at a concentration of 606 μg ml^−1^ was added to give a final probe concentration of 6 μg ml^−1^. Samples were incubated at 25°C in the dark for 10 min before being exposed to heat stress (or analysis in the case of the unstressed control).

To stain samples after the thermal treatment, 10 μl of heat-treated and untreated (control) yeasts were mixed with 40 μl of sterile PBS prepared in Millipore Milli-Q (0.22 μm filtered) water and 50 μl of propidium iodide stock solution at a concentration of 12 μg ml^−1^ to give a final probe concentration of 6 μg ml^−1^. Samples were incubated at 25°C in the dark for 10 min before analysis.

DiBAC_4_(3) was used to determine the impact of the heat treatments on the membrane potential of the yeast cells. Of heat-treated or untreated (control) yeast cells, 100 μl was mixed with 0.58 μl of DiBAC4(3) stock solution at a concentration of 1 mM to give a final probe concentration of 5.7 μM. Samples were incubated at 25°C in the dark for 10 min before being analyzed.

### Statistical analysis

When referred to in figure legends, the means and the 95% confidence intervals of the means of at least four independent measurements were calculated. Group means were compared using the two-tailed unpaired Student’s *t*-test performed by matlab® software (R2011b; 7.13.0.564 version). Groups were analysed by performing ttest2 function. The null hypothesis was that the means were equal and the alternative hypothesis was that means were not equal. In both cases, the *p-value* (*P*) was calculated. Significance was accepted at *P* < 0.05.
